# Supplementary material for: Genome-wide somatic variant calling using localized colored de Bruijn graphs
Source: Commun Biol. 2018 Mar 22;1:20. doi: 10.1038/s42003-018-0023-9 (PMC6123722; doi:10.1038/s42003-018-0023-9)
Supplement: Supplementary file 1 — Supplementary Information(PDF 3605 kb) [file 42003_2018_23_MOESM1_ESM.pdf]

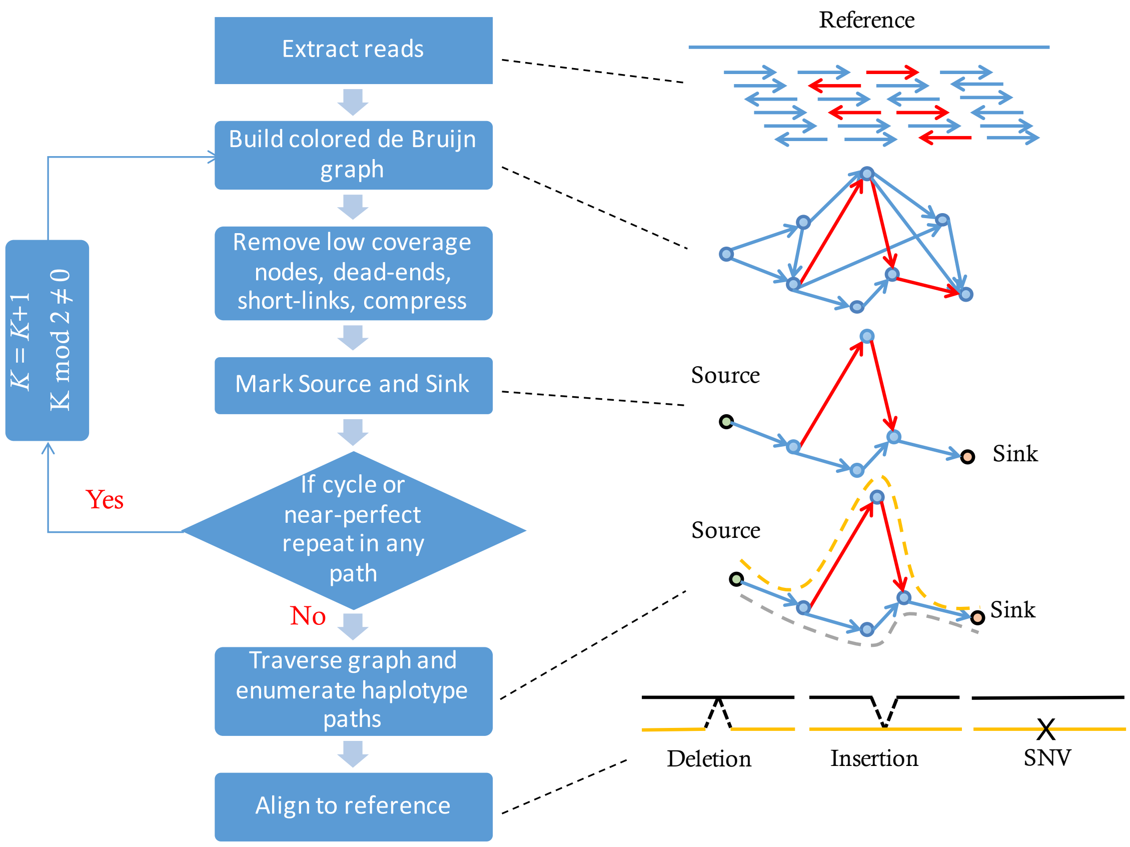

Supplementary Figure 1. Lancet workflow. Local assembly workflow employed by Lancet to assemble each genomic window. Extracted reads from the tumor and the normal samples are decomposed into *k*-mers and used to build a colored DeBruijn graph. The *k*-mer size is automatically tuned to avoid the presence of perfect and near-perfect repeats in the graph. Only odd values of *k* are used to avoid *k*-mers which are reverse complement of their own sequence. Standard graph transformations are applied to reduce the graph complexity. Source and sink nodes are selected to anchor the graph to the reference window and then explored to extract source-to-sink paths. The assembled sequences are finally aligned to a reference to identify the mutations using the standard Smith-Waterman-Gotoh alignment algorithm with affine-gap penalties.

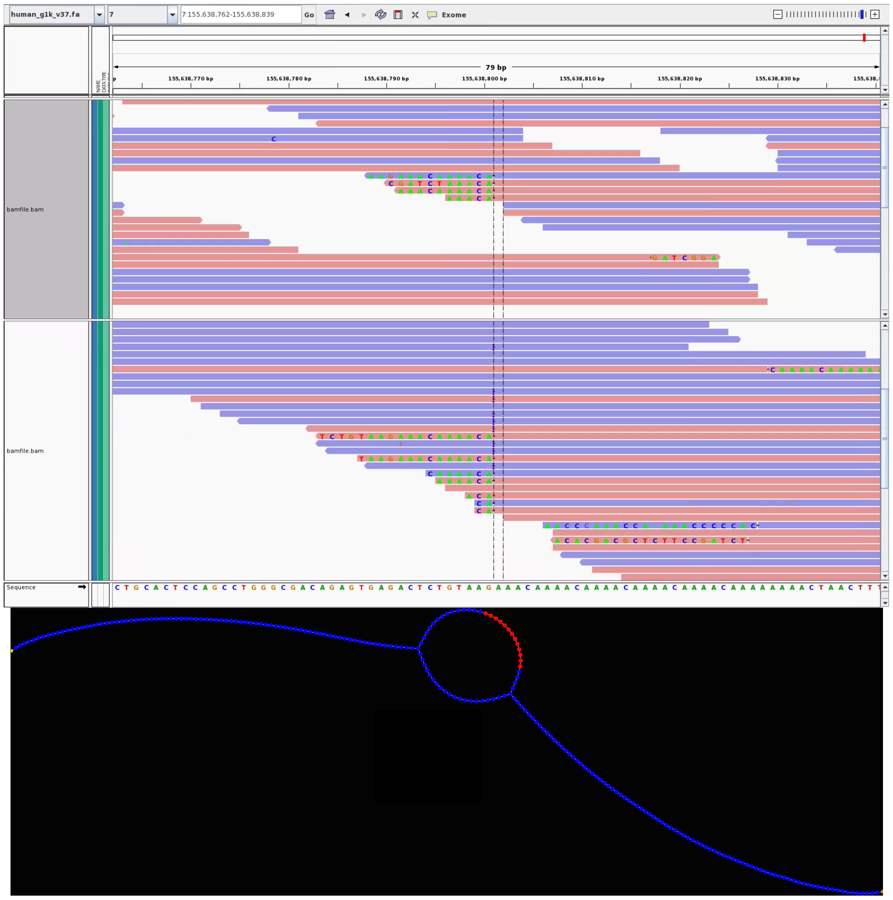

Supplementary Figure 2. Example of partially supported bubble. (top) IGV snapshot of an insertion present in the tumor by inspecting the alignments. The same mutation is partially supported in the normal as marked by the matching soft-clipped sequences in the normal and tumor reads. The combination of low support in the normal (soft-clipping) and the presence of low complexity sequence in the reference, confounds the aligner and affects variant allele fraction estimations based on the mapped reads. **(bottom)** The colored DeBruijn graph of the tumor and normal reads shows a partially supported bubble, which correctly characterizes the mutation as shared between the normal and tumor samples. Moreover, the *k*-mers counts along the bubble allow accurate estimation of the variant allele fraction.

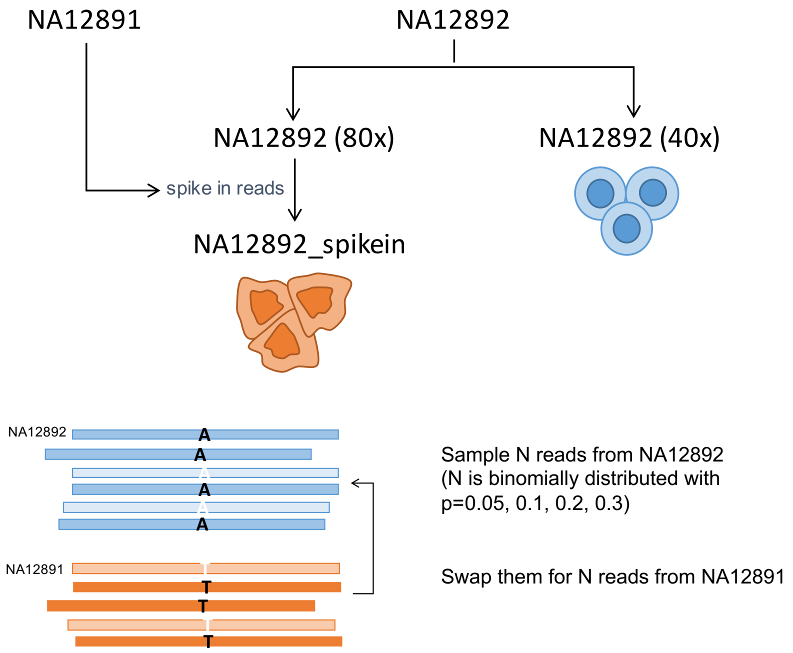

Supplementary Figure 3. Virtual tumors approach. **(top)** Reads from sample NA12892 are randomly partitioned into two subsets of 80x and 40x average coverage corresponding to the tumor and normal samples respectively. A second samples (NA12891) is used to spike in mutations (SNVs and indels) in the 80x coverage subset of the data. **(bottom)** Mutations are spiked in by swapping a predefined number of reads between the two samples at specific loci where NA12892 is homozygous reference and NA12891 is homozygous alternative. By controlling the number N of reads that are swapped (e.g., binomial distribution with mean $\mu = 0.05$, 0.1, 0.2, 0.3) we insert mutations at any desired variant allele fraction.

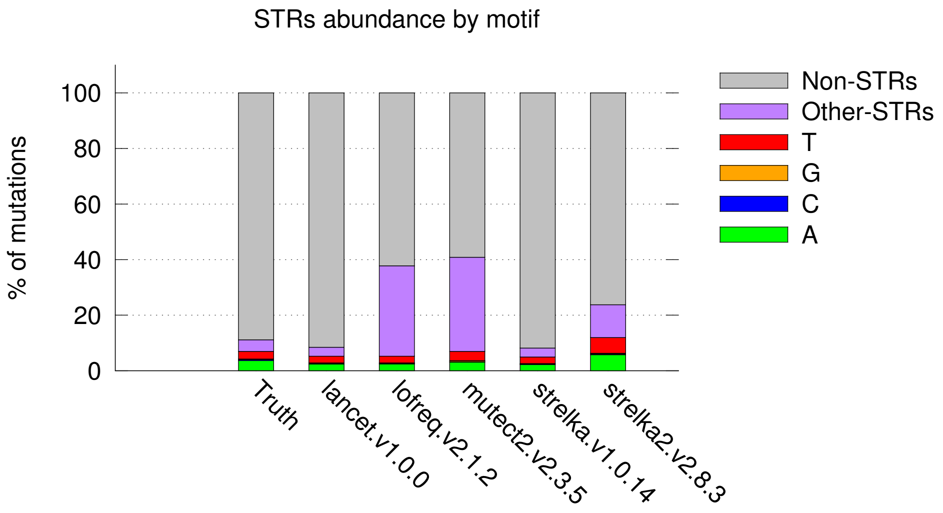

Supplementary Figure 4. Short tandem repeat abundance on the virtual tumor. Percentage of short tandem repeats in the truth calls set and in the somatic variants called by lancet, LoFreq, MuTect2, Strelka, and Strelka2.

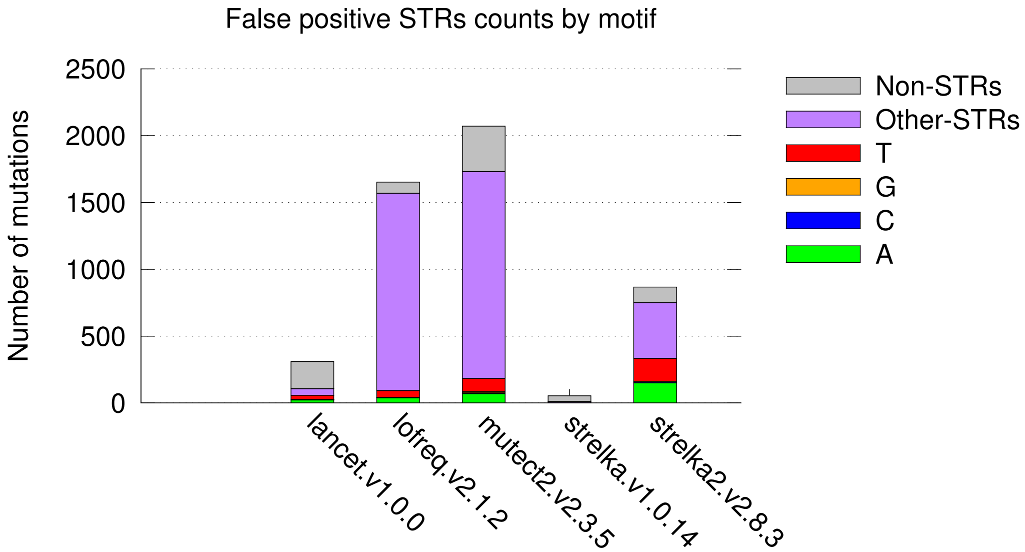

Supplementary Figure 5. False positive STR indel counts. Number of false positive indels by motif within short tandem repeats (STR) for each somatic caller (lancet, LoFreq, MuTect2, Strelka, and Strelka2) in the virtual tumor analysis. STRs are defined as sequences composed of at least 7bp (total length), where the repeat sequence is between 1bp and 4bp, and is repeated at least 3 times. Homopolymers are reported separately for each base pair (A,C,G,T), while other STRs, whose motif is composed of more than one single base, are grouped together.

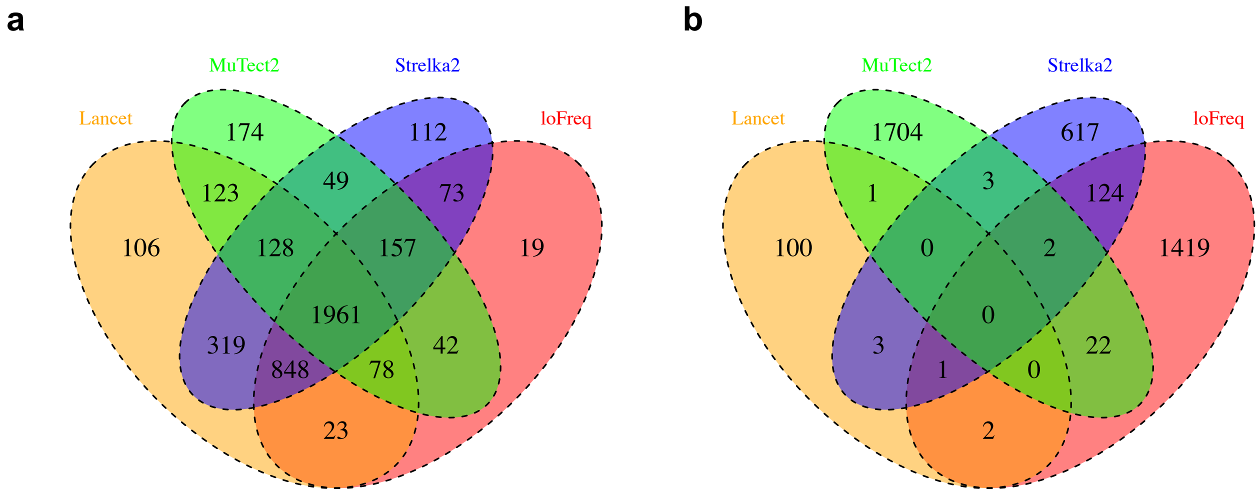

Supplementary Figure 6. Indel Venn diagrams for the virtual tumor. Venn diagrams of the number of true positive indels **(a)** and false positive STR indels **(b)** for Lancet, MuTect2, Strelka2, and LoFreq on the virtual tumor.

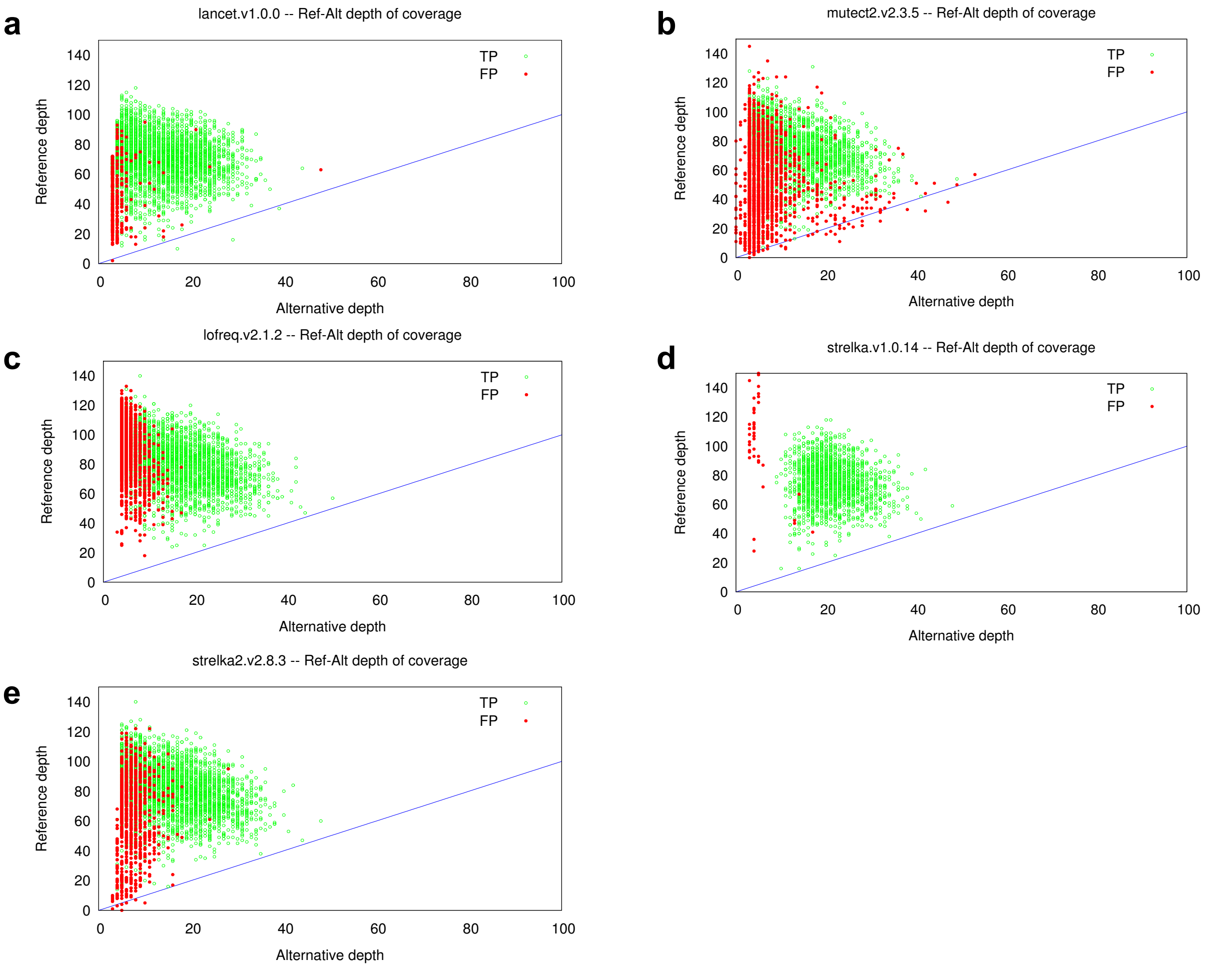

Supplementary Figure 7. Reference and alternative coverage depths of indels called in the virtual tumor. Number of supporting reads for the reference and alternative somatic allele as reported by **(a)** Lancet, **(b)** MuTect2, **(c)** LoFreq, **(d)** Strelka, and **(e)** Strelka2 in the virtual tumor.

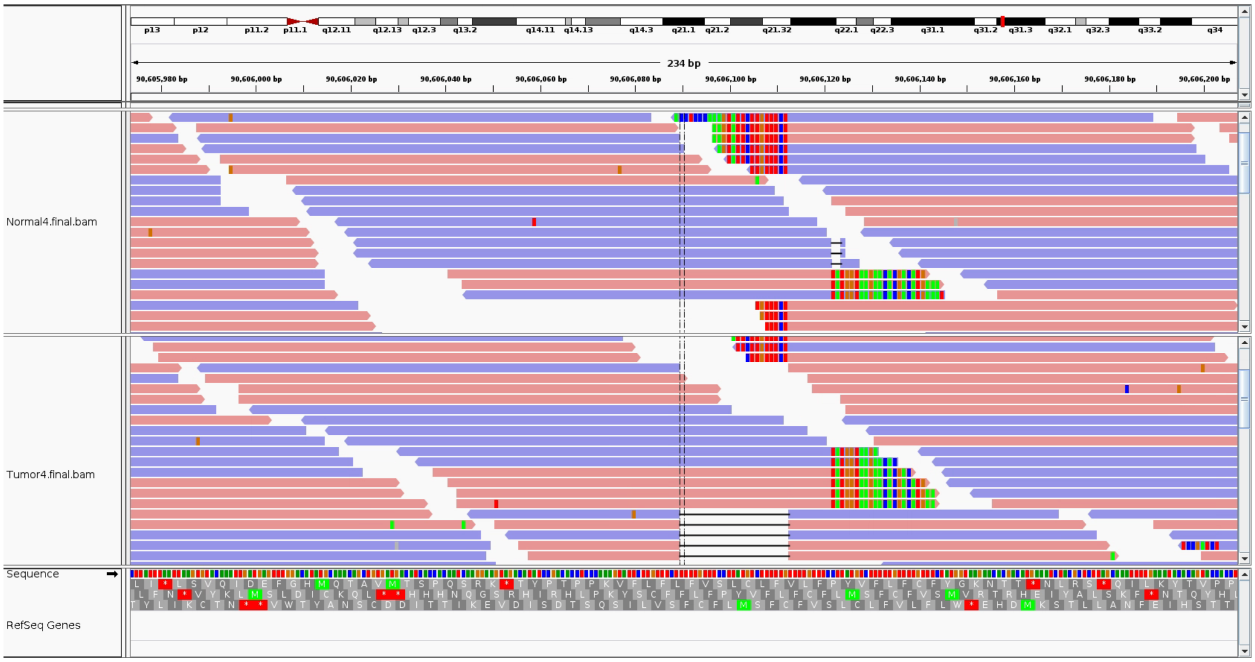

Supplementary Figure 8. IGV snapshot of a false positive LoFreq indel. Illustrative example of one of the false positive indels reported by LoFreq on the ICGC-TCGA DREAM data synthetic challenge #4. Mis-alignment of the reads in the normal sample prevents the tool from correctly classifying this mutation as germline.

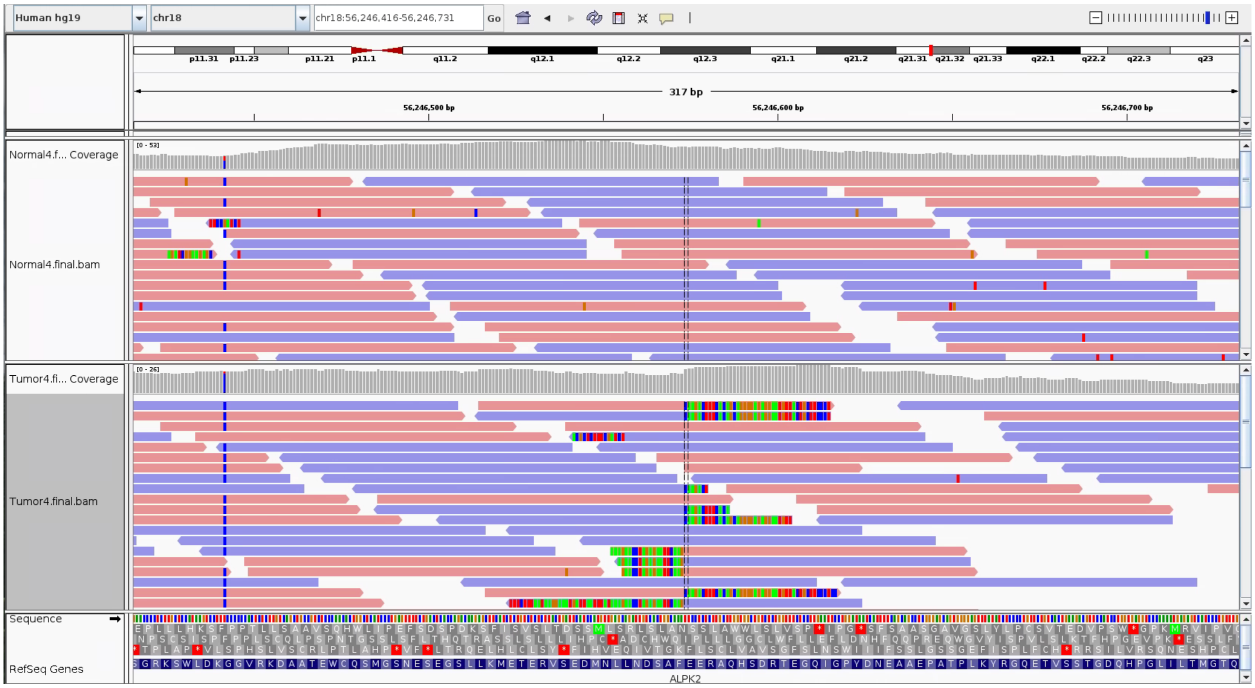

Supplementary Figure 9. IGV snapshot of a false positive MuTect2 indel. Illustrative example of one of the false positive indels reported by MuTect2 on the ICGC-TCGA DREAM data synthetic challenge #4. Soft-clipped reads signal the presence of a mutation in the tumor, but those reads belong to only one of the two breakpoints of a much a larger structural event, inversion of 5697 base pairs (**Supplementary Fig. 15**), that is mis-classified as a short insertion of 29 base pairs.

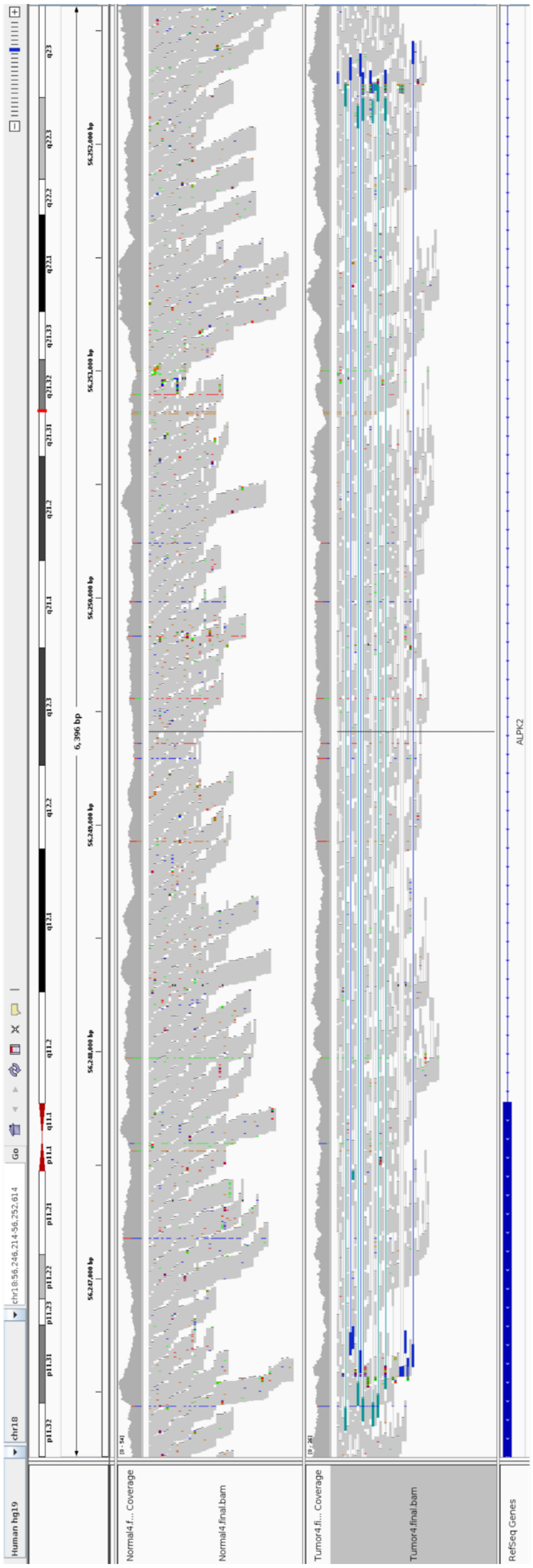

Supplementary Figure 10. IGV snapshot of a large inversion in the ICGC-TCGA DREAM data. IGV Illustration of a large inversion of 5697 base pairs on the ICGC-TCGA DREAM data synthetic challenge #4 with soft-clipped reads signal at both breakpoints and discordant read pairs. Blue read pairs are both aligned in forward orientation (→ →), green read pairs are both aligned in reverse orientation (← ←).

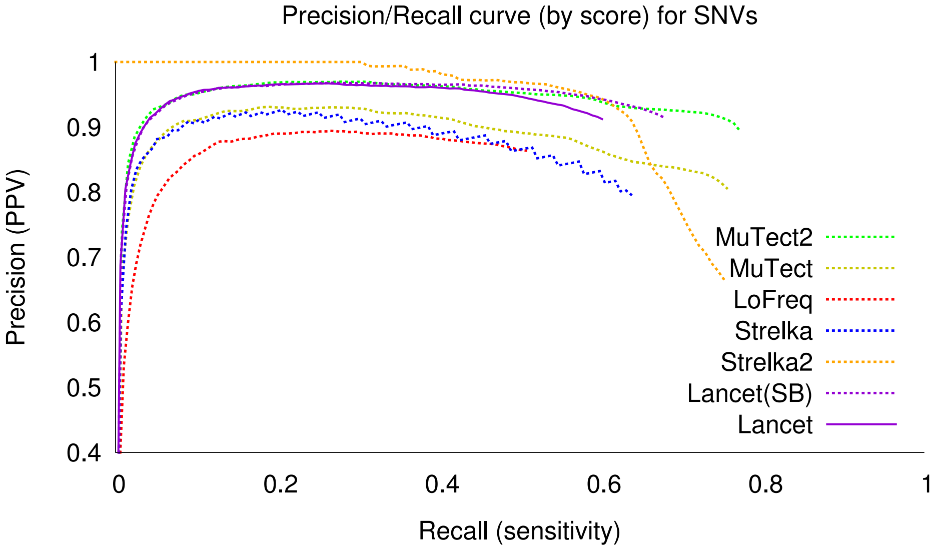

Supplementary Figure 11. Precision/recall SNV performance on the DREAM data synthetic challenge #4. Precision/recall curve analysis of somatic SNVs called by Lancet, MuTect, MuTect2, LoFreq, Strelka, and Strelka2 on the Synthetic challenge #4 of the ICGC-TCGA DREAM mutation calling challenge. Lancet^{SB} is the version of Lancet run with strand bias filter turned off. Curves are generated by sorting the mutations based on the confidence score assigned by each tool (from highest quality to lowest). Each point of the curve corresponds the precision and recall for all the variants with confidence score greater than or equal to a specific quality threshold. The curve for an ideal tool (no errors) would start from the top left corner and produce a straight horizontal line (with precision=1). Any deviation from a straight line is due to errors introduced by the variant calling process. Specifically, deviations at low recall rates are indicative of low performance of the scoring system adopted by the tool (false positive variants with high score).

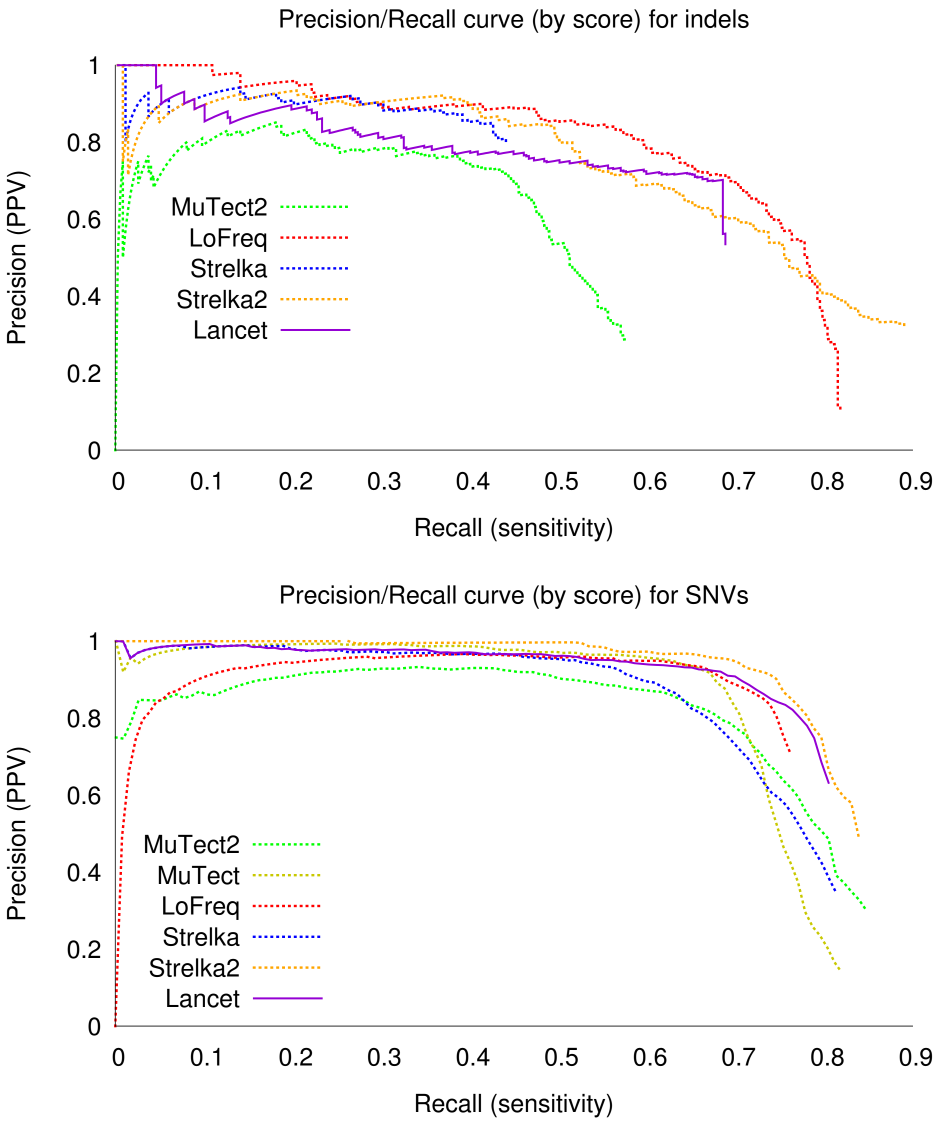

Supplementary Figure 12. Precision/recall performance on the ICGC medulloblastoma tumor-normal pair. Precision/recall curve analysis of somatic indels **(top)** and SNVs **(bottom)** called by Lancet, MuTect, MuTect2, LoFreq, Strelka, and Strelka2 on the ICGC medulloblastoma tumor-normal pair. Curves are generated by sorting the mutations based on the confidence score assigned by each tool (from highest quality to lowest). Each point of the curve corresponds the precision and recall for all the variants with confidence score greater than or equal to a specific quality threshold. The curve for an ideal tool (no errors) would start from the top left corner and produce a straight horizontal line (with precision=1). Any deviation from a straight line is due to errors introduced by the variant calling process. Specifically, deviations at low recall rates are indicative of low performance of the scoring system adopted by the tool (false positive variants with high score).

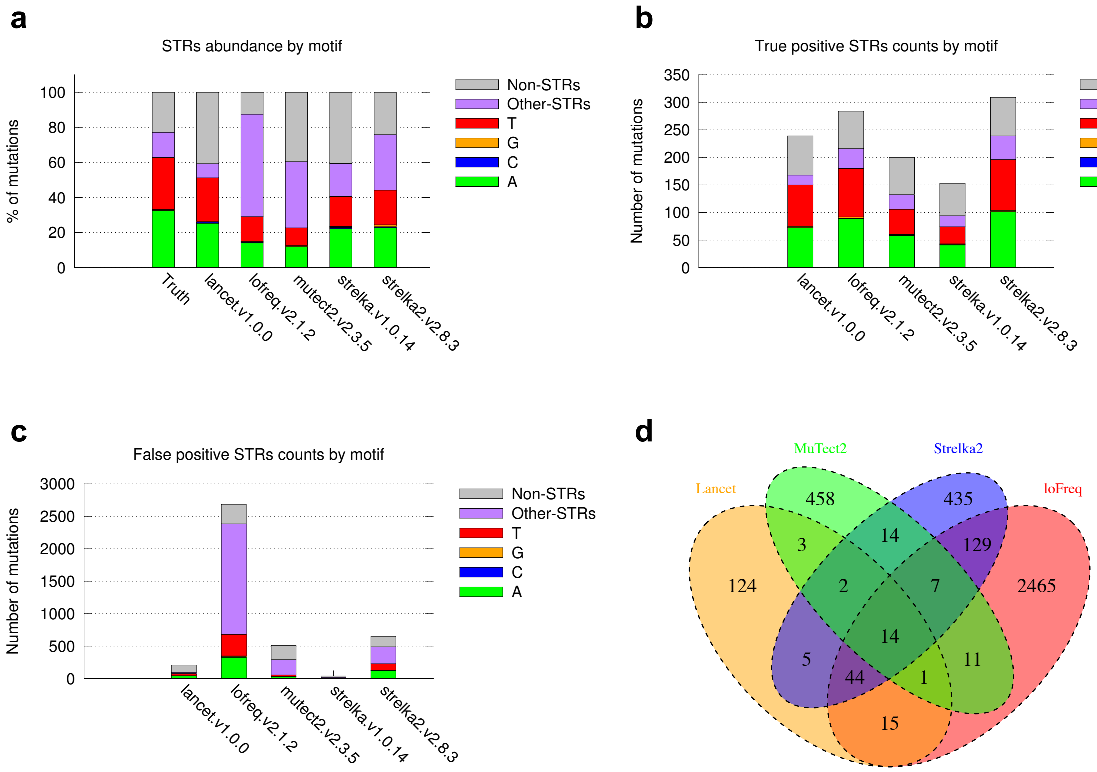

Supplementary Figure 13. False positive STR indels called in the medulloblastoma data. (a) Percentage of short tandem repeats (STR) in the truth calls set and in the somatic variants called by Lancet, LoFreq, MuTect2, Strelka, and Strelka2. **(b)** Number of true positive indels called by each tool classified according to their STR motif. **(c)** Number of false positive indels by motif within STRs for each somatic variant caller. **(d)** Venn diagrams of the number of false positive indels within STRs for Lancet, MuTect2, Strelka2, and LoFreq. Short tandem repeats are defined as sequences composed of at least 7bp (total length), where the repeat sequence is between 1bp and 4bp, and is repeated at least 3 times. Homopolymers are reported separately for each base pair (A,C,G,T), while other STRs whose motif is composed of more than one single base are grouped together.

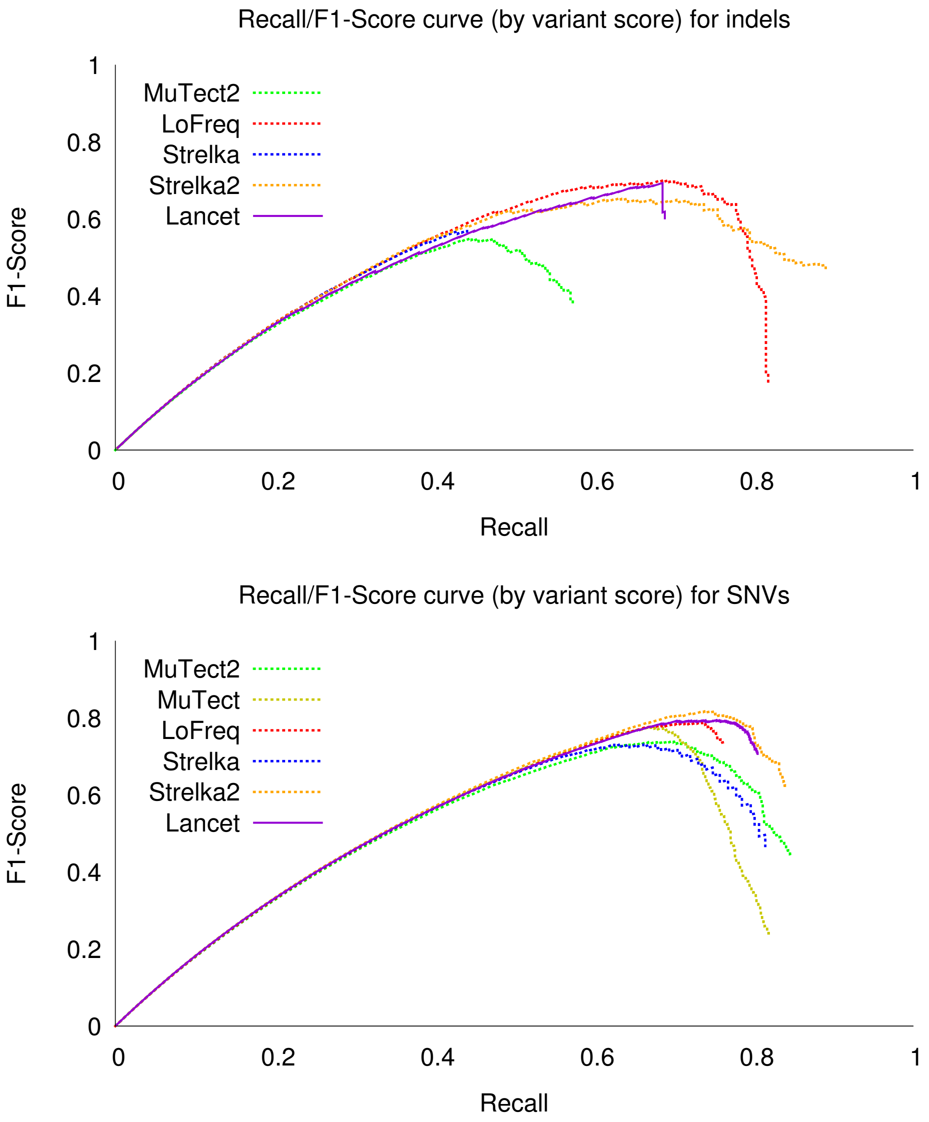

Supplementary Figure 14. F_1-score/recall curve on the ICGC medulloblastoma tumor-normal pair. F_1-score is plotted as a function of recall for somatic indels **(top)** and SNVs **(bottom)** called by Lancet, MuTect, MuTect2, LoFreq, Strelka, and Strelka2 on the ICGC medulloblastoma tumor-normal pair. Curves are generated by sorting the mutations based on the confidence score assigned by each tool (from highest quality to lowest). Each point of the curve corresponds to the F_1-score and recall pair for all the variants with confidence score greater than or equal to a specific quality threshold. The maximum point of the curve along the Y axis, the maximum F_1-score, corresponds to the optimal F_1-score for any given variant quality threshold cutoff, and it can be interpreted as the best possible performance of a tool when filtering variants according to their quality. The F_1-score is defined as the harmonic mean of precision and recall: 2×(precision×recall)/(precision+recall).

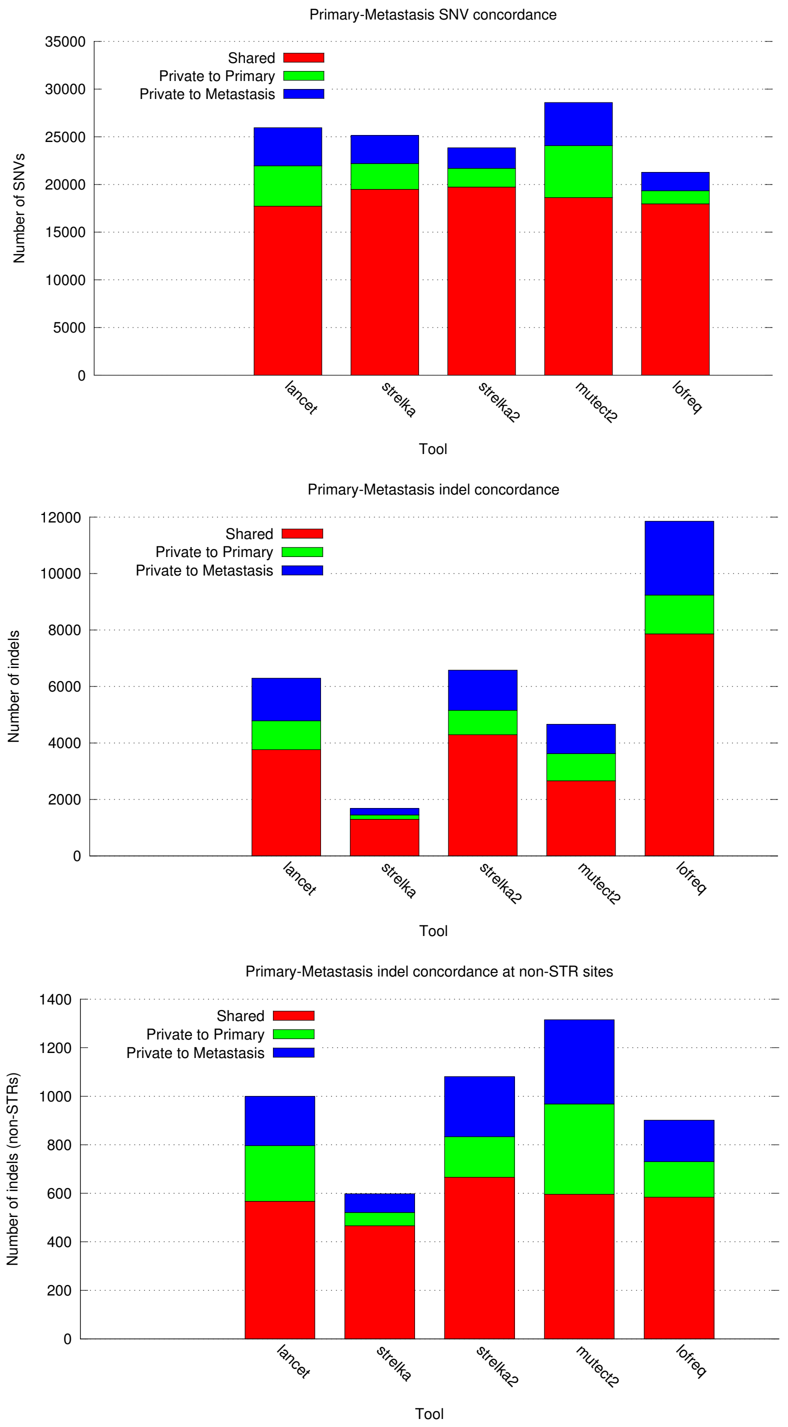

Supplementary Figure 15. Whole-genome mutational concordance between primary and metastasis for a pair of highly genetically concordant colorectal cancer samples. Number of SNVs **(top)**, indels **(center)**, and non-STR indels **(bottom)** shared or private to primary and metastasis as detected by Lancet, LoFreq, MuTect2, Strelka, and Strelka2. The number of shared indels is more variable among the tools compared to the SNVs.

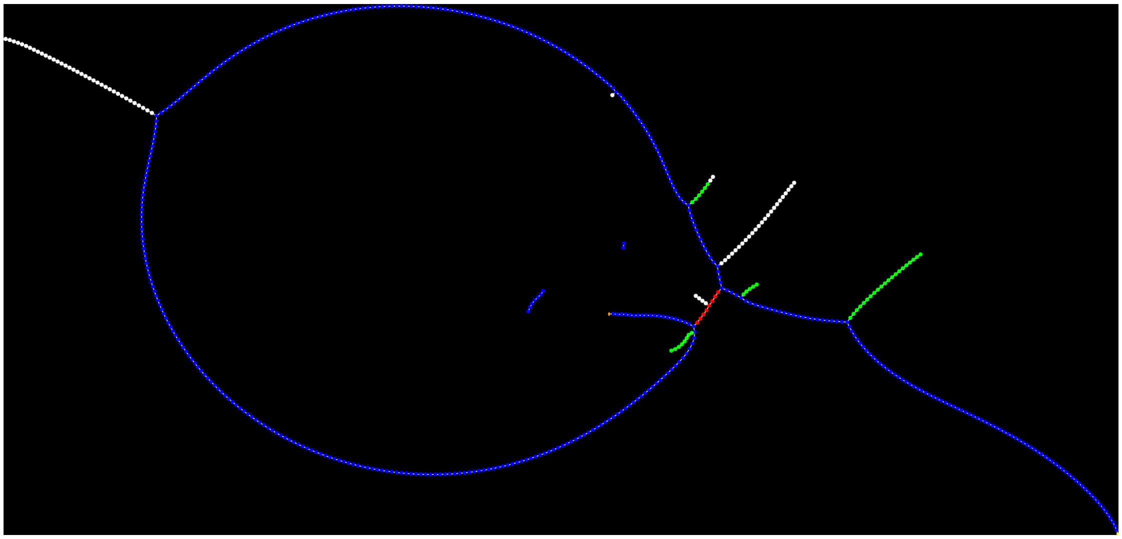

Supplementary Figure 16. **Example of colored DeBruijn graph containing a short-link.** Example of a large bubble (no cycles) where a short-link introduces a spurious connection which causes a false-positive large deletion to be called in the tumor. Blue nodes correspond to *k*-mers shared by both the tumor and the normal samples, red nodes correspond to *k*-mers private to the tumor, green nodes correspond to *k*-mers private to the normal, and white nodes correspond to low coverage *k*-mers due to sequencing errors.

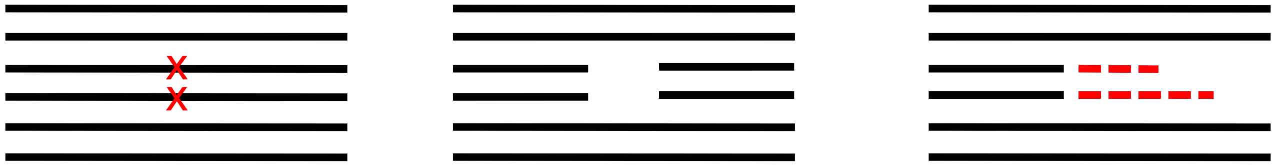

Supplementary Figure 17. Active regions policy. A region is "active", and will be processed to discover variants using local-assembly, if either in the tumor or the normal sample there is a minimum of *N* (aligned) reads supporting a mismatch, indel, or soft-clipped sequence at the same locus.

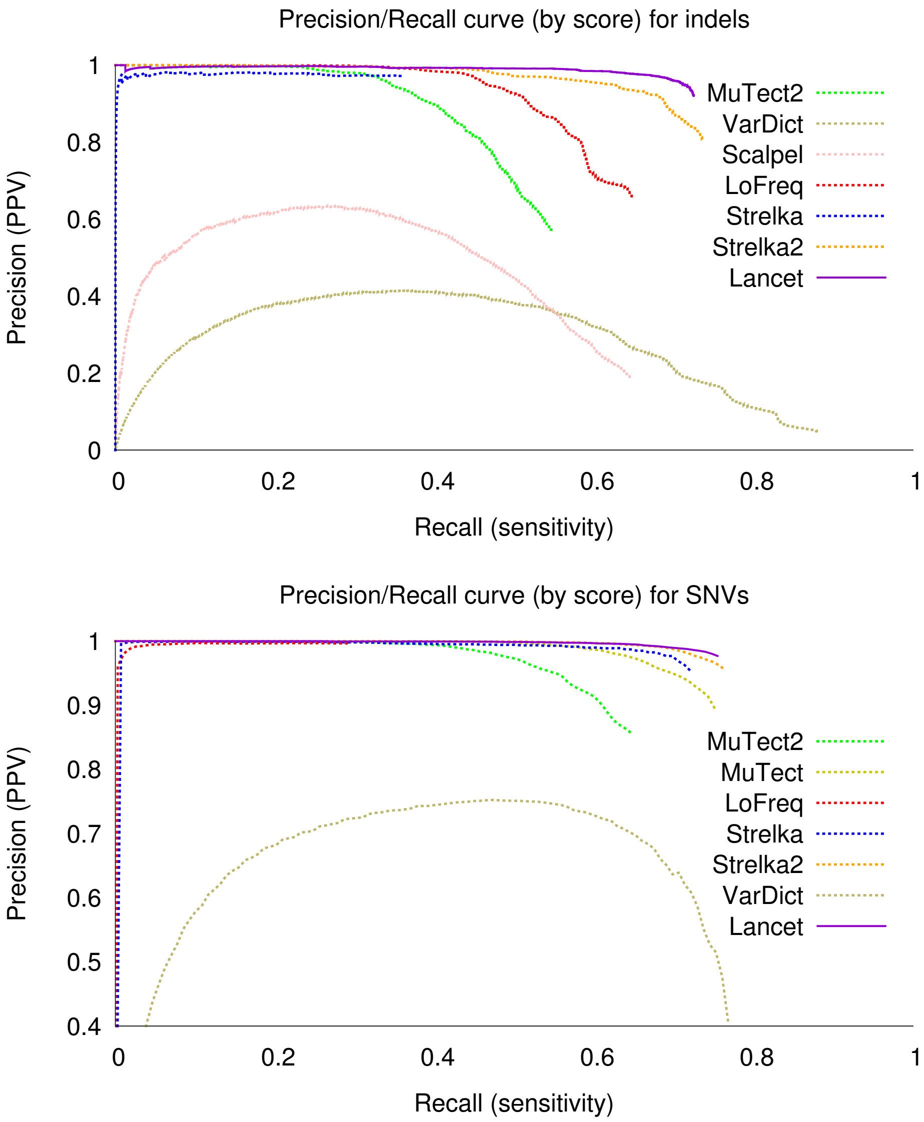

Supplementary Figure 18. Precision/recall performance on the virtual tumors for an extended number of somatic variant callers. Precision/recall curve analysis of somatic indels **(top)** and SNVs **(bottom)** called by Lancet, MuTect, MuTect2, LoFreq, Strelka, Strelka2, Scalpel, and VarDict on the ICGC medulloblastoma tumor-normal pair. Curves are generated by sorting the mutations based on the confidence score assigned by each tool (from highest quality to lowest). Each point of the curve corresponds the precision and recall for all the variants with confidence score greater than or equal to a specific quality threshold. The curve for an ideal tool (no errors) would start from the top left corner and produce a straight horizontal line (with precision=1). Any deviation from a straight line is due to errors introduced by the variant calling process. Specifically, deviations at low recall rates are indicative of low performance of the scoring system adopted by the tool (false positive variants with high score).

Supplementary Table 1. Somatic indel detection performance on the on the ICGC-TCGA DREAM data synthetic challenge #4. Tools sorted in descending order of F_1-score. Lancet^{SB} is run with strand bias filter turned off. Bold entries represent the best performing tool on the associated metric.

	# of calls	True Positive	False Positive	False Negative	Recall	Precision	FDR	F_1score[a]	Max F_1score[b]
**Lancet^{SB}**	10943	10642	251	3588	0.75	0.97	0.023	**0.85**	**0.85**
**MuTect2**	11711	11052	659	3178	**0.77**	0.94	0.056	**0.85**	**0.85**
**Lancet**	8523	8483	40	5747	0.59	**0.99**	**0.004**	0.74	0.74
**Strelka2+Manta**	12207	9360	2847	4870	0.65	0.76	0.23	0.70	0.70
**Strelka2**	10834	7992	2842	6238	0.56	0.73	0.26	0.63	0.63
**LoFreq**	14765	7443	7322	6787	0.52	0.50	0.495	0.51	0.51
**Strelka**	1175	1157	18	13073	0.081	0.98	0.015	0.15	0.15

[a] F_1score: harmonic mean of precision and recall, 2×(precision×recall)/(precision+recall); [b] Maximum F_1score computed for each combination of precision and recall along the precision/recall curve.

Supplementary Table 2. Somatic SNV detection performance on the on the ICGC-TCGA DREAM data synthetic challenge #4. Tools sorted in descending order of F_1-score. Lancet^{SB} is run with strand bias filter turned off. Bold entries represent the best performing tool on the associated metric.

	# of calls	True Positive	False Positive	False Negative	Recall	Precision	FDR	F_1score[a]	Max F_1score[b]
**MuTect2**	14117	12611	1506	3704	**0.77**	0.89	0.106	**0.83**	**0.83**
**Lancet^{SB}**	12056	11041	1015	5274	0.68	**0.92**	**0.084**	0.78	0.78
**MuTect**	15308	12338	2970	3977	0.76	0.81	0.194	0.78	0.78
**Lancet**	10835	9820	945	6495	0.60	0.91	0.087	0.73	0.73
**Strelka2**	18440	12276	6164	4039	0.75	0.67	0.334	0.71	0.75
**Strelka**	13201	10402	2799	5913	0.64	0.79	0.212	0.7	0.71
**LoFreq**	9609	8283	1326	8032	0.51	0.86	0.137	0.64	0.64

[a] F_1score: harmonic mean of precision and recall, 2×(precision×recall)/(precision+recall); [b] Maximum F_1score computed for each combination of precision and recall along the precision/recall curve.

Supplementary Table 3. Somatic indel detection performance on the ICGC medulloblastoma tumor-normal pair. Tools sorted in descending order of F_1-score. Bold entries represent the best performing tool on the associated metric.

	# of calls	True Positive	False Positive	False Negative	Recall	Precision	FDR	F_1score[a]	Max F_1score[b]
**Lancet**	447	239	208	108	0.69	0.53	0.46	**0.60**	0.69
**Strelka**	192	153	39	194	0.44	**0.79**	**0.20**	0.56	0.57
**Strelka2**	959	309	650	38	**0.89**	0.32	0.67	0.47	0.65
**MuTect2**	710	200	510	147	0.57	0.28	0.71	0.37	0.55
**LoFreq**	2970	284	2686	63	0.81	0.09	0.90	0.17	**0.70**

[a] F_1score: harmonic mean of precision and recall, 2×(precision×recall)/(precision+recall); [b] Maximum F_1score computed for each combination of precision and recall along the precision/recall curve.

Supplementary Table 4. Somatic SNV detection performance on the ICGC medulloblastoma tumor-normal pair. Tools sorted in descending order of F_1-score. Bold entries represent the best performing tool on the associated metric.

	# of calls	True Positive	False Positive	False Negative	Recall	Precision	FDR	F_1score[a]	Max F_1score[b]
**LoFreq**	1360	962	398	301	0.76	**0.71**	**0.29**	**0.73**	0.78
**Lancet**	1614	1017	597	246	0.81	0.63	0.36	0.71	0.79
**Strelka2**	2191	1060	1131	203	0.84	0.48	0.51	0.61	**0.81**
**Strelka**	3136	1029	2107	234	0.81	0.33	0.64	0.47	0.73
**MuTect2**	3582	1071	2511	192	**0.85**	0.30	0.70	0.44	0.74
**MuTect**	7522	1036	6486	227	0.82	0.14	0.86	0.24	0.77

[a] F_1score: harmonic mean of precision and recall, 2×(precision×recall)/(precision+recall); [b] Maximum F_1score computed for each combination of precision and recall along the precision/recall curve.

Supplementary Table 5. Computational requirements for each tool when analyzing the 80x/40x sequencing data of the virtual tumor.

	Variant type	Calling paradigm	Core hours	Max memory (GB)
**Lancet**	SNVs & indels	Pure local-assembly	2580	18
**MuTect2**	SNVs & indels	Hybrid: assembly+alignment	700	10
**LoFreq**	SNVs & indels	Alignment-based	464	7
**Strelka**	SNVs & indels	Alignment-based	72	2
**Strelka2**	SNVs & indels	Alignment-based	28	1.2
**MuTect**	SNVs	Alignment-based	345	8
